# Intention, Motivations, and Barriers to Emigration of Nursing Students in Colombia

**DOI:** 10.17533/udea.iee.v42n3e13

**Published:** 2024-10-29

**Authors:** Bairon Steve Peña-Alfaro, Nancy Viviana Torres Díaz

**Affiliations:** 1 Nurse, Ph.D. Assistant Professor, Faculty of Nursing - Universidad Nacional de Colombia. Email: bspenaa@unal.edu.co. Corresponding author. https://orcid.org/0000-0002-3963-9069 Universidad Nacional de Colombia Faculty of Nursing Universidad Nacional de Colombia Colombia bspenaa@unal.edu.co; 2 Nurse, Master’s. Quality Auditor, RTS S.A.S. Bogotá-Colombia. Email: nvtorresd@unal.edu.co. https://orcid.org/0000-0002-4966-732X RTS S.A.S RTS S.A.S Bogotá Colombia

**Keywords:** emigration and immigration, nursing students, nursing schools, health workforce, working conditions, Colombia., emigración e inmigración, estudiantes de enfermería, facultades de enfermería, fuerza laboral en salud, condiciones de trabajo, Colombia., emigração e imigração, estudantes de enfermagem, escolas de enfermagem, mão de obra no sector da saúde, condições de trabalho, Colômbia.

## Abstract

**Objective.:**

To explore the intention, motivations, and barriers to emigrate of final semester nursing students from Colombia.

**Methods.:**

Quantitative, descriptive, and cross-sectional study with participation by 556 last-semester students matriculated in 26 undergraduate nursing programs in Colombia. Data were collected through an online questionnaire.

**Results.:**

The study found that 84% of the participants consider among their plans as future nursing professionals to emigrate to practice their profession in another country. Destinations of preference for those who have thought of emigrating include countries, like Canada (63.5%), Spain (57.7%), Germany (44.9%), and the United States (44.4%). The main reasons that motivate nursing students to emigrate when they complete their professional studies are: better remuneration (81.6%), better quality of life (67.9%), greater professional growth (64.1%), greater job stability (54.7%), and more employment options (49.8%). In turn, the reasons that discourage nursing students from emigrating when they complete their professional studies are: language (71.9%), going away from the family (60.6%), and the complexity of the emigration process (55.4%).

**Conclusion.:**

The findings of this research show that a notable proportion of last-semester nursing students consider among their plans to emigrate to practice in another country when they receive their degree. Knowing the intentions, motivations, and barriers to emigrate of future nurses will permit having elements to design strategies that improve the retention of professionals in Colombia.

## Resumen

**Objetivo.:**

Explorar la intención, motivaciones y barreras para emigrar de estudiantes de último semestre de enfermería de Colombia.

**Métodos.:**

Estudio cuantitativo, descriptivo y de corte transversal con participación de 556 estudiantes de último semestre matriculados en 26 programas de pregrado de Enfermería en Colombia. Los datos se recolectaron a través de un cuestionario en línea.

**Resultados.:**

El 84 % de los participantes considera dentro de sus planes como futuros profesionales de enfermería emigrar para ejercer la profesión en otro país. Los destinos de preferencia para quienes han pensado emigrar incluyen países como Canadá (63.5%), España (57.7%), Alemania (44.9%) y Estados Unidos (44.4%). Las principales razones que motivan a los estudiantes de enfermería a emigrar cuando culminen sus estudios profesionales son: mejor remuneración (81.6%), mejor calidad de vida (67.9%), mayor crecimiento profesional (64.1%), mayor estabilidad laboral (54.7%) y más opciones de empleo (49.8%). Por su parte, las razones que desmotivan a los estudiantes de enfermería a emigrar cuando culminen sus estudios profesionales son: el lenguaje o idioma (71.9%), alejarse de la familia (60.6%) y la complejidad del proceso para emigrar (55.4%).

**Conclusión.:**

Los hallazgos de esta investigación muestran que una apreciable proporción de los estudiantes de enfermería de último semestre consideran dentro de sus planes emigrar para ejercer en otro país cuando cuenten con su título. El conocimiento de las intenciones, motivaciones y barreras para emigrar de los futuros enfermeros permitirá tener elementos para el diseño de estrategias que mejoren retención de los profesionales en Colombia.

## Resumo

**Objetivo.:**

Explorar a intenção, motivações e barreiras para emigrar de estudantes de enfermagem no último semestre da Colômbia.

**Métodos.:**

Estudo quantitativo, descritivo e transversal com a participação de 556 estudantes finalistas matriculados em 26 cursos de graduação em Enfermagem na Colômbia. Os dados foram coletados por meio de questionário online.

**Resultados.:**

84% dos participantes consideram emigrar para exercer a profissão noutro país como parte dos seus planos enquanto futuros profissionais de enfermagem. Os destinos preferidos de quem já pensou em emigrar incluem países como Canadá (63.5%), Espanha (57.7%), Alemanha (44.9%) e Estados Unidos (44.4%). Os principais motivos que motivam os estudantes de enfermagem a emigrarem quando concluem os seus estudos profissionais são: melhor remuneração (81.6%), melhor qualidade de vida (67.9%), maior crescimento profissional (64.1%), maior estabilidade profissional (54.7%). mais opções de emprego (49.8%). Por sua vez, os motivos que desencorajam os estudantes de enfermagem de emigrar quando concluem os estudos profissionais são: a língua (71.9%), o afastamento da família (60.6%) e a complexidade do processo de emigração (55.4%).

**Conclusão.:**

Os resultados desta investigação mostram que uma proporção significativa de estudantes de enfermagem no último semestre considera emigrar para exercer a profissão noutro país como parte dos seus planos quando obtiverem a licenciatura. O conhecimento das intenções, motivações e barreiras à emigração dos futuros enfermeiros fornecerá elementos para o desenho de estratégias que melhorem a retenção de profissionais na Colômbia.

## Introduction

In a globalized environment, such as the current, and with differences that seem to be important for nurses in terms of remuneration, working conditions, and the social value of their profession, emigration ends up becoming a life alternative for many. For Colombia, the nursing practice is precarious, poorly recognized at social and institutional levels and, given its particularities, it is a quite emotionally and physically demanding profession.^1^ It is likely that this is perceived by undergraduate nursing students and, in the face of possibilities of a better future, they consider the alternative of emigrating to practice in another country once they have their degree.

The World Health Organization has expressed concern because of the increased global deficit of health personnel and the imbalance between the offer, demand, and the needs of said personnel.^2^ In the particular case of nursing, the number of foreign nurses that have been received in developed countries has increased by 60% since 2010. Migration is accompanied by a shortage of around 20.7-million nurses and midwives around the world,^3^ a situation that increased with the Covid-19 pandemic. In a survey by the International Council of Nurses (ICN) in December 2020 of national associations of nurses, one of every five of them reported an increase in the number of professionals who abandoned the practice as consequence of the pandemic.^4^ It has been suggested that poor working conditions of nurses in developing countries lead them to emigrate to more developed countries,^5^ although migration to other countries can imply that the nurse who emigrates becomes exposed to situations of labor exploitation.^6^ For the region of Latin America, the migratory phenomenon of nurses between 2006 and 2011 had as principal host countries Brazil, which received 35.1% of the migrants, Venezuela 22.6%, Chile 21.7%, and Argentina 14.6%, while the principal countries of origin of the migrants were Peru, which represented 95.1% of nurses emigrating from the region, followed by Paraguay with 2.6%.^7^

Immigrants bring human and financial capital to the host country and, at the same time, bring to their country of origin not only economic resources but also the knowledge, skills, and cultural elements learned from the host country. In addition, migration is recognized as strongly linked to the development of countries and regions.^8^ However, for the countries of origin, it represents a loss of human capital that affects the health system not only due to the reduction of staff *per se*, but also because of the compromise on the quality of care, which increases inequality. Furthermore, if professionals cannot perform as such in the host country, it is required that upon re-entering the country of origin they be prepared to practice the profession there, with the economic and time costs that this implies.^9^

In the scientific literature available, few recent studies are found on the intention to emigrate in nursing. Some of them, conducted in Asian and European countries, explored the intention to emigrate by nurses and advanced-level nursing students and the reasons or factors to do so.^10^^-^^15^ Nevertheless, no studies carried out in Colombia related to this phenomenon were identified, which is why the research question arose: what is the intention, motivations, and barriers to emigrate of last-semester students from undergraduate nursing programs in Colombia? According with the foregoing, the aim of this research was to explore the intention, motivations, and barriers to emigrate of last-semester students from undergraduate nursing programs in Colombia. It is expected for this study to contribute to understand a little-explored phenomenon that is decisive in the construction of human resource policies in nursing for Colombia.

## Methods

Study design. This was a cross-sectional study with a quantitative approach and descriptive scope carried out between February and April 2024. 

Population. Last-semester students registered in 26 undergraduate nursing programs from 23 university institutions in Colombia. The total of possible participating students registered in the last semesters in these 26 nursing programs was 819 students for the first academic period of 2024. All the possible students were invited to participate, obtaining a final sample of 556 participants, for a participation percentage of 67.8%, using non-probabilistic sampling. 

Instrument. The study used a questionnaire elaborated by the researchers from the scientific literature available. The questionnaire had three sections: (i) sociodemographic questions, (ii) questions about the intention to emigrate, plans and preparation; and (iii) motivations and barriers to emigrate. Once the questionnaire was elaborated, a pilot test was carried out with participation from 17 last-semester undergraduate nursing students from two university institutions. During this test, each participant was asked about the clarity, ease of comprehension, and ease to answer each of the questions. With the data obtained, an acceptance index was calculated for each question, where values close to 1 indicated acceptance by the majority of the participants. Values were obtained between 0.94 and 1 in terms of clarity, between 0.82 and 1 in ease of comprehension, and between 0.88 and 1 in ease of response. From the results of the pilot test, the questionnaire was consolidated for its application. 

Collection of information. The questionnaire was incorporated onto the secure Research Electronic Data Capture (REDCap) software platform to collect research study data and, through a hyperlink, it was disclosed to the academic units (Nursing faculties, departments, schools) from the university institutions that accepted to support the research and who directly forwarded the hyperlink to the students; or, in other cases, the hyperlink was allowed to be sent to the students directly by the research team via e-mail. Periodically, reminders were sent to the academic units to encourage student participation and completion of the questionnaire.

Data analysis. Data was extracted directly from the REDCap platform, then it was organized and incorporated onto the SPSS statistical software for analysis. The results were obtained from the use of descriptive statistics, particularly frequencies and percentages. 

Ethical considerations. This research received approval 003-23 from the Research Ethics Committee of the Faculty of Nursing at Universidad Nacional de Colombia. In compliance with the ethical principles of research, informed consent for participation was included in the REDCap platform, where participants were informed of the objective of the research, the purpose of their participation, and the possibility of withdrawing at any time without any repercussions, along with the appropriate treatment that would be given to the data by the research team with the relevant anonymity and confidentiality, for which reason, personal data that would allow identifying the participants was not included in the form. The consent had to be accepted by the participants prior to their filling out the form.

## Results

The study obtained 675 completed forms, of which 3 (0.4%) were discarded due to not providing consent to participate, 101 (14.9%) due to incomplete information, and 15 (2.2%) because participants were not in the final enrollment of their undergraduate nursing program. Thus, the results of this research were generated from 556 forms completely filled out by students from the last academic semester of 23 undergraduate nursing programs in Colombia.

The principal demographic characteristics of the participants included mean age of 24.5 ± 4.2 years (minimum 20 years and maximum 40 years); the highest percentage were of female biological sex (82%); 98.9% had Colombian nationality by origin and, of those remaining, five were born in Venezuela and had Colombian nationality and one was born in Spain without having Colombian nationality. Regarding marital status, 84.9% of the participants were single, 9.9% were in a common-law relationship, 4.5% were married, 0.4% were divorced, and 0.4% were widowed. In addition, 15.8% of the students had children (1 = 11.7%, 2 = 3.8% and 3 = 0.4%). The study also found that 27.1% of the students knew other countries and 70.5% had relatives residing outside of Colombia.

The participants resided in 20 departments of the country, with Cundinamarca (16.1%), Valle del Cauca (15.8%), and Santander (12.4%) being the departments with the highest participation of students; 58% of the participants indicated being in the last enrollment of their professional nursing studies in a private university institution and the rest were from a public university institution.

To find out about the participants' communication skills in other languages, they were required to select from a list of languages ​​that they read, wrote, and spoke, other than Spanish. Only 30.4% reported reading, speaking, and writing at least one language different from Spanish; with English being the most frequent in nine of every ten cases.

With respect to the intention to emigrate, the participants were asked if among their plans as future professionals they have considered emigrating to practice the profession in another country, to which 84% of the participants responded affirmatively (of these, 56.6% from private university institutions versus 43.4% from public university institutions).

The participants who have considered emigrating (*n =* 468) to practice their nursing profession in another country when they have their professional degree were asked to select from a list a maximum of three of their interest. Canada is the country most-often selected by the participants (*n =* 297), followed by Spain (*n =* 270), Germany (*n =* 210), the United States (*n =* 208), the United Kingdom (*n =* 70), Mexico (*n =* 56), Argentina (*n =* 44), Chile (*n =* 38), and Portugal (*n =* 14). Other destinations selected by the participants were: Italy (*n =* 18), Australia (*n =* 15), Brazil (*n =* 7), the Netherlands (*n =* 4), Sweden (*n =* 4), Austria (*n =* 3), Switzerland (*n =* 3), France (*n =* 3), Japan (*n =* 3), Poland (*n =* 2), Norway (*n =* 2), South Korea (*n =* 1), El Salvador (*n =* 1), New Zealand (*n =* 1), Israel (*n =* 1), Panama (*n =* 1), United Arab Emirates (*n =* 1), Belgium (*n =* 1),and Luxemburg (*n =* 1).

The 468 participants who expressed their intention to emigrate were also asked about the activities or requirements they have carried out or are carrying out in order to emigrate, with the most frequent being: searching for information about employment and professional practice in the destination country (52.1%), taking courses to learn another language (49.4%), requesting information about requisites to arrive at the destination country (36.5%), contacting with people or companies that provide advice and accompany emigration (21.8%), preparing for general or profession-specific knowledge tests required by the destination country (11.8%), and contacting with possible employers (11.1%) (Table 1). 

**Table 1:** Activities currently carried out to be able to emigrate (*n =* 468)

Activities				Frequency	Percentage
Search for information about employment and professional practice in the destination country.	Has not begun	224	47.9
	Is doing so	226	48.3
	Already completed it	18	3.8
Request for information about requisites to arrive at the destination country (visa, passport, homologation or recognition of degrees, work permit, etc.)	Has not begun	297	63.5
	Is doing so	156	33.3
	Already completed it	15	3.2
Contact with people or companies that provide advice and accompany emigration of nursing professionals	Has not begun	335	71.6
	Is doing so	97	20.7
	Already completed it	5	1.1
	Not considered necessary	31	6.6
Contact with possible employers	Has not begun	416	88.9
	Is doing so	48	10.3
	Already completed it	4	0.9
Request for information of documents (certificates or evidence) in the university where they study to initiate homologation procedures abroad.	Has not begun	416	88.9
	Is doing so	47	10.0
	Already completed it	5	1.1
Language learning course.	Has not begun	186	39.7
	Is doing so	193	41.2
	Already completed it	38	8.1
	Does not apply	51	10.9
Preparation for knowledge tests (general or profession-specific) required by the destination country.	Has not begun	395	84.4
	Is doing so	50	10.7
	Already completed it	5	1.1
No proficiency tests are required	18	3.8

With regards to the motivations to emigrate of the participants who have considered practicing the nursing profession in another country when they get their degree (*n =* 468), the main reasons can be observed in Table 2, thus: better remuneration (81.6%), better quality of life for me (67.9%), greater professional growth (64.1%), more job stability (54.7%), and more employment options (49.8%).

**Table 2:** Reasons that motivate emigrating to practice the profession in other countries (*n =* 468)

Reason	Frequency	Percentage
Better remuneration	382	81.6
Better quality of life for me	318	67.9
Greater professional growth	300	64.1
More job stability	256	54.7
More employment options	233	49.8
Being able to provide better education and/or quality of life for the family	184	39.3
Better professional recognition	162	34.6
Greater professional satisfaction	154	32.9
Knowing a different culture	146	31.2
Possibility of studying at graduate level	144	30.8
Knowing the professional practice in another country	92	19.7
More personal security (less violence, for example)	89	19
Better work environment	83	17.7
Having new links	69	14.7
Reuniting with relatives	28	6
Accompanying a relative who has job security in that country	14	3

Finally, all the participants (*n =* 556) were asked about the principal reasons that can discourage or limit the intention of emigrating to other countries to practice the nursing profession. According with Table 3, the principals reasons reported include language (71.9%), going away from the family (60.8%), and the complexity of the emigration process (55.4%). It was also possible to identify among the other reasons the lack of financial resources to carry out the process and procedures required to emigrate (2%).

**Table 3:** Reasons that discourage emigrating to practice the profession in other countries (*n =* 556)

Reasons	Frequency	Percentage
Language	400	71.9
Going away from the family	338	60.8
The complexity of the emigration process	308	55.4
Differences in the professional practice among countries	191	34.4
The possibility of finding good employment in Colombia	162	29.1
Difference in culture	149	26.8
Differences in health technology among countries	98	17.6
Going away from Friends	93	16.7
Lack of skill and competence to perform as a nurse	82	14.7
Other reasons	11	2

## Discussion

The results herein permitted recognizing that emigrating is an attractive option for students who are about to finish their university undergraduate nursing studies in Colombia. For recent graduates and professionals alike, facing the reality of professional practice can differ from their expectations as students and from their role as such,^16^ being an expected reason to consider the option of emigrating. However, this study found that, even without having faced professional life in Colombia, last-semester students set their expectations on the professional practice abroad and, in addition, have plans to do so.

The high percentage of respondents motivated to emigrate, > 80%, exceeds that found in Mexico, where the intention to emigrate to work by students was 65%.^17^ Studies conducted in other countries, like Nepal, Serbia, Ghana, Indonesia, and Ireland have also evidenced the intention to emigrate of nursing students.^18^ According to health statistics for 2021 by the Organization for Economic Cooperation and Development ​ (OECD), which compared the number of nurses per one-thousand inhabitants between 2011 and 2021, it was found that, among the countries evaluated, the third country with the lowest number of nurses is Colombia with (1.6 x 1000 inhabitants).^19^ Thus, the greatest implication of this interest would undoubtedly lie in that, if the emigration of these future nursing professionals were to materialize, their increased deficit in Colombia would result in a threat to the Colombian health system and in a public health problem of enormous magnitude. Moreover, the deficit of nursing professionals could motivate health institutions to seek strategies to meet the needs of professional services with nursing aides, affecting the quality and security of nursing care provided to the Colombian population. 

The destination countries selected to emigrate in this study show how the spectrum of these destinations broadens from countries in America to Europe, Asia, and Oceania. Germany, selected by almost half of the respondents, is a country that has made cooperation efforts to bridge the significant shortage of nurses in their country with Colombian nurses and, in this task, it is accompanied by some university institutions and private companies that prepare their students for such. Canada, Spain, Germany, and the United States lead the group of countries to emigrate to among the respondents; three of them have also been sought after by Mexican students,^17^ with the United States and Canada being two of the four destinations of greater interest for Asian students, together with Australia and the United Kingdom.^11^^,^^13^ Interest also exists in emigrating to Latin American countries, like Mexico, Argentina, and Chile, which have one less requirement to access them: that of language. Interest in these destinations reveals how migratory movements are established in Latin America from “south to south”, that is, between the countries of the region, and not “south to north” as is usually expected, from developing countries to developed countries.^20^

According to the density of nurses in practice for every one-thousand inhabitants published by the OECD^19^ and the students’ destinations of preference in this study, it is possible to indicate that, if their emigration is carried out in the future, Colombia would be “exporting” nursing professionals to countries, like Germany and the United States that have 10.4 more nurses for every 1000 inhabitants than Colombia; Canada with 8.7 more nurses for every 1000 inhabitants than Colombia; and Spain with 4.7 more nurses per 1000 inhabitants than Colombia. This would increase the shortage of nurses in Colombia to an unsustainable point for the health system, which would consequently increase inequality in access to nursing care in the country. 

Special attention should be paid to the emigration intention of students who are currently being trained in programs whose funding source is public resources from the State to supply the deficiencies and needs of human talent specific to the country. If their emigration were to materialize, State investment would be used to meet the human talent needs of other countries. Several of the respondents who are interested in emigrating are making concrete plans to do so or have already completed them, especially in relation to finding information about the destination country, contacting companies looking to hire nurses, and - very specially - preparing themselves in the language required by the destination country, a task being carried out or already completed by half of them. 

Regarding plans to emigrate, the work of recruiting companies to find nurses is of particular concern, given experiences of nurses who have emigrated through these companies and who ended up being exploited in what has been called "co-ethnic exploitation" when it is compatriots who exploit nurses from the beginning of recruitment to labor exploitation in the destination country.^21^ Of course, with or without intermediaries, emigrating entails risks of discrimination, as has been documented from studies carried out in Australia and the United Kingdom,^22^^,^^23^ although these studies do not include Latin American nurses.

Among the motivations to emigrate, remuneration is the most frequent, being the factor that has been recognized as central to emigrate in other studies.^10^^,^^15^^,^^24^^,^^25^ According to a report on the health labor market in Colombia, with data from 2022, a nursing professional earns monthly $2 325 344 Colombian pesos (Approx. $607USD - Exchange Rate 17/05/2024) for working an average of 48 hours per week, at a rate of cop $47 748 per hour.^26^ Although the income of Colombian nurses is above the national average, it does not seem to be compatible with the responsibilities, physical effort, mental exhaustion of the nursing professionals, nor with the hours of work required to carry out their work.

Professional growth is another aspect considered relevant for the respondents, as found in a multicenter study conducted with Italian nurses.^12^ The interest of young nursing students in achieving a better quality of life is striking, given that this has been recognized as a motivating force to emigrate,^10^^,^^24^^,^^27^ which is related with the equilibrium of life at work and life outside of work. The respondents in this study possibly perceive instability in the employment of nursing professionals in Colombia, which coincides with the results presented by the Labor Observatory at Universidad del Rosario from an analysis of the Large Integrated Household Survey that found that 38.7% of nursing professionals do not have indefinite labor contracts.^26^ With respect to the barriers to emigrate, the principal discouraging factor was language, which is consistent with the activities being undertaken currently to emigrate, where most of the participants are taking a course to learn another language. Among the other factors, the reference to the complexity of the emigration process is striking, reported by more than half of the respondents. Added to this is the fact that some students were explicit in pointing out economic reasons as another barrier when considering this option, as documented in the literature on the subject.^28^

Conclusion. Emigrating is an option of interest for 84% of the participants who are close to completing undergraduate university nursing studies in Colombia. These results show government entities, like the ministries of health and labor, the need to implement measures aimed at controlling the control the so-called brain drain that could be occurring in nursing. The fact that students express intention to emigrate suggests that government entities, nursing professional associations and unions should urgently work on retaining the new generations of professionals to prevent the migration phenomenon from worsening the shortage of nurses in the country, leading to a public health crisis. 

It will not be enough to train more nursing professionals if, from those responsible for human talent in health and nursing leaders, there is no work on aspects related to remuneration, job stability, and quality of life of nurses because, eventually, the country would end up training an appreciable number of professionals who will end up practicing the profession outside of Colombia. Due to the aforementioned, it is essential that, in the country's political agenda, the government establish measures and strategies to regulate the emigration and immigration of human talent in health, including nursing professionals who opt for this life alternative and professional practice. It is considered pertinent and necessary to continue researching this phenomenon, replicating these types of studies in Latin America to identify the emigration intention of current and future nursing professionals and, thus, design strategies and key interventions to improve professional retention in the country. 

The principal limitation of this study is the impossibility of generalizing the results to the entire population of last academic semester undergraduate nursing students in Colombia. Nevertheless, the strategies used to achieve participation permitted having an important number of students. 

## References

[1] Consejo Técnico Nacional de Enfermería (2013). Declaración del Consejo Técnico Nacional de Enfermería [Internet]. Encolombia.com.

[2] Organización Mundial de la Salud 69a Asamblea Mundial de la Salud. Estrategia mundial de recursos humanos para la salud: personal sanitario 2030 [Internet]. Who.int.

[3] Zolot J (2019). International nurse migration. American Journal of Nursing.

[4] Consejo Internacional de Enfermeras (2021). Resumen de evidencia para políticas del Consejo Internacional de Enfermeras. Escasez mundial de enfermería y retención de enfermeras.

[5] Li H, Nie W, Li J (2014). The benefits and caveats of international nurse migration. International Journal of Nursing Sciences.

[6] Van den Broek D, Groutsis D (2017). Global nursing and the lived experience of migration intermediaries. Work, Employment and Society [Internet].

[7] Organización Panamericana de la Salud (2011). Migración de enfermeras en América Latina-Área de América del Sur. Serie Recursos Humanos para la Salud.

[8] International Organization for Migration (IOM), International Labour Organization (ILO), United Nations Development Programme (UNDP) (2016). Sociology of migration and development. Supporting evidence for thematic area 2, Ninth Global Forum on Migration and Development, Bangladesh.

[9] Fernández Aranda M. I (2018). Efectos de la migración de enfermeras y matronas en los sistemas sanitarios europeos. Matronas Hoy.

[10] Hendel T, Kagan I (2011). Professional image and intention to emigrate among Israeli nurses and nursing students. Nurse Education Today.

[11] Lee E, Moon M (2013). Korean nursing students’ intention to migrate abroad. Nurse Education Today.

[12] Palese A, Falomo M, Brugnolli A, Mecugni D, Marognolli O, Montalti S (2017). Nursing student plans for the future after graduation: a multicentre study. International Nursing Review.

[13] Poudel C, Ramjan L, Everett B, Salamonson Y (2018). Exploring migration intention of nursing students in Nepal: A mixed-methods study. Nurse Education in Practice.

[14] Deasy C, O Loughlin C, Markey K, O Donnell C, Murphy Tighe S, Doody O (2021). Effective workforce planning: Understanding final-year nursing and midwifery students’ intentions to migrate after graduation. Journal of Nursing Management.

[15] Efendi F, Oda H, Kurniati A, Hadjo SS, Nadatien I, Ritonga IL (2021). Determinants of nursing students’ intention to migrate overseas to work and implications for sustainability: The case of Indonesian students. Nursing & Health Sciences.

[16] Mozolová V, Tupá M (2024). Migration intentions of nurses and nursing students from Slovakia: A study on drivers. Problems and Perspectives in Management.

[17] Rosales-Martínez Y, Nigenda G, Galárraga O, Ruiz-Larios JA (2010). Expectativas de migración internacional en estudiantes de enfermería en México, Distrito Federal. Salud Pública de México.

[18] Tosunöz İK, Nazik E (2022). Career future perceptions and attitudes towards migration of nursing students: A cross-sectional multicenter study. Nurse Education in Practice.

[19] Organization for Economic Co-operation and Development (OECD) (2023). "Practicing nurses per 1 000 population, 2011 and 2021 (or nearest year)", in: Health at a Glance 2023: OECD Indicators, OECD Publishing, Paris.

[20] Ivkovic M (2011). International nurse migrations: Global trends. Journal of the Geographical Institute “Jovan Cvijić” SASA.

[21] Velayutham S (2013). Precarious experiences of Indians in Australia on 457 temporary work visas. The Economic and Labour Relations Review.

[22] Batnitzky A, McDowell L (2011). Migration, nursing, institutional discrimination and emotional/affective labour: ethnicity and labour stratification in the UK National Health Service. Social & Cultural Geography.

[23] Smith JB, Herinek D, Woodward-Kron R, Ewers M (2022). Nurse migration in Australia, Germany, and the UK: A rapid evidence assessment of empirical research involving migrant nurses. Policy, Politics, & Nursing Practice.

[24] Jadhav ST, Roy SM (2024). The sociodemographic characteristics of Indian nursing students and their intentions to migrate overseas for work. Policy, Politics, & Nursing Practice.

[25] Konlan KD, Lee TW, Damiran D (2023). The factors that are associated with nurse immigration in lower‐ and middle‐income countries: An integrative review. Nursing Open.

[26] García A, Holguín C, Ríos J, Rodríguez P, Romero B (2023). Radiografía del mercado laboral para los profesionales de la salud.

[27] Oda H, Tsujita Y, Irudaya Rajan S (2018). An analysis of factors influencing the international migration of Indian nurses. Journal of International Migration and Integration.

[28] Milasi S (2020). What drives youth’s intention to migrate abroad? Evidence from international survey data. IZA Journal of Development and Migration.

